# Dupuytren’s Contraction of the Fingers

**Published:** 1882-07

**Authors:** 


### DUPUYTREN'S CONTRACTION OF THE FINGERS.

Dr. W. W. Keen (Philadelphia *Medical Times*, March 11, 1882), after a very careful and exact examination of one hundred and twenty-six cases of this disease coming under his observation, and found in the literature of whom one hundred and six were males and twenty females, finds that of forty-four cases exactly recorded, twenty-eight began after forty, nine between thirty and forty, and only seven before thirty; of these seven, two were certainly and a third probably congenital. Out of seventy-two cases, eighteen were persons having manual, and fifty-four of persons having non-manual, occupations. The two hands were attacked in nearly equal proportion, both hands being affected in thirty-six out of seventy-two cases. The three ulnar fingers are the greatest sufferers. Heredity was noted in one third the cases. Gout, hereditary or personal, was noted in forty-two out of forty-eight cases. Taking everything into consideration, Dr. Keen is inclined to believe that traumatism is not, as has been alleged, a principal cause, but such cause is to be found in a constitutional vice, like rheumatism or gout, which seems to produce a species of predisposition which produces the contraction.—*Chic. Med. Rev.*

